# α^+^Thalassemia Antagonizes the Malaria-protective Effects of Sickle-Cell Trait

**DOI:** 10.1016/j.ebiom.2014.10.020

**Published:** 2014-10-30

**Authors:** Chanaki Amaratunga, Rick M. Fairhurst

**Affiliations:** Laboratory of Malaria and Vector Research, National Institute of Allergy and Infectious Diseases, National Institutes of Health, Rockville, MD 20852, USA

In 2012, an estimated 207 million malaria cases and 627,000 malaria deaths were reported worldwide (World Health Organization, 2013). For millennia, *Plasmodium falciparum* malaria has exerted significant evolutionary pressure on the human genome, selecting for balanced polymorphisms that confer disease protection. This possibility was first proposed in 1949 by J. B. S. Haldane, who suggested that reduced fitness in *β*-thalassemia homozygotes due to severe anemia was offset by increased fitness in heterozygotes due to *P. falciparum* malaria protection (Haldane, 1949). In 1954, A. S. Allison demonstrated in Kenya that hemoglobin S (HbS) heterozygosity (HbAS, sickle-cell trait) conferred protection from malaria, and proposed that this protection balanced the severe anemia morbidity and premature fatality associated with HbS homozygosity (HbSS, sickle-cell disease) (Allison, 1954). Clinical studies have since associated HbC and α^+^thalassemia with malaria protection (Williams, 2006, Taylor et al., 2012), and shown that α^+^thalassemia antagonizes HbAS; that is, HbAS children who co-inherit α^+^thalassemia are not protected from malaria (Williams et al., 2005).

While the mechanisms by which hemoglobinopathies confer malaria protection are debated (Taylor et al., 2013), with the notable exception of HbAS, they do not seem to protect by suppressing parasite densities. Epidemiological observations, together with in-vitro data, support alternative mechanisms of protection that enable individuals to “tolerate” parasitemia without developing symptoms. In one mechanism, HbS and α^+^thalassemia each impairs binding of parasitized red blood cells (pRBCs) to microvascular endothelial cells (MVECs, cytoadherence) and uninfected RBCs (rosetting), two virulence traits mediated by interactions between *P. falciparum* erythrocyte membrane protein 1 (PfEMP1) ligands on pRBCs and various receptors (CD36, ICAM1, endothelial protein C receptor [EPCR]) on host cells. We have always believed that future experimental data consistent with this mechanism are more likely to be biologically and clinically relevant if they reconcile with robust epidemiological findings.

In this issue of *EBioMedicine*, Opi et al. (2014) meet exactly this type of criterion in proposing a mechanism to explain their original 2005 report of negative epistasis between α^+^thalassemia and HbAS in Kenya (Williams et al., 2005). Specifically, they hypothesized that if α^+^thalassemia antagonizes malaria-protective effects of HbAS in the field, then it should also antagonize cytoadherence-weakening effects of HbAS in the laboratory. Using RBCs from individuals with HbAS, α^+^thalassemia, or both, they performed experiments that measure cytoadherence, rosetting, and PfEMP1 expression of four *P. falciparum* lines. While their reductionist approach of using few parasite strains is a minor limitation of this study, they made up for this by using a large panel of RBCs from hemoglobinopathic donors (10–20 of each genotype) (Opi et al., 2014). Their findings largely confirm earlier reports that HbAS and α^+^thalassemia are each associated with impaired cytoadherence, rosetting, and PfEMP1 expression (Cholera et al., 2008, Krause et al., 2012), with two exceptions. For one parasite strain, α^+^thalassemia increased PfEMP1 expression without affecting rosetting; for another, α^+^thalassemia increased PfEMP1 expression but paradoxically decreased cytoadherence.

These findings contrast with those of Krause et al. (2012), who generally found that α^+^thalassemia reduced both PfEMP1 expression and cytoadherence (α^+^thalassemia increased the cytoadherence of *some* parasite isolates) (see Fig. 1A–B of reference Krause et al., 2012). These apparent discrepancies may result from marked differences in the authors' use of (i) antibodies specific for PfEMP1 variants expressed by parasite lines ITvar9 and TM284var1 (vs. FVO, A4tres, and FCR3^CSA^); (ii) four laboratory-adapted parasite lines (vs. multiple naturally-circulating parasite isolates from Malian children with malaria); and (iii) purified CD36 and ICAM1 proteins as binding substrates (vs. CD36-expressing MVECs and monocytes). As Opi et al. (2014) emphasize, future studies should test large numbers of both RBC donors and *P. falciparum* strains to help resolve these differences, which will hopefully provide more consistent experimental data that adequately explain well-established field observations in Africa (reviewed in Taylor et al., 2012). These differences notwithstanding, the major new finding of this study is that α^+^thalassemia consistently reverses HbAS-mediated reductions in cytoadherence, rosetting, and PfEMP1 expression in vitro.

This solid result now provides impetus for exploring how α^+^thalassemia antagonizes the effects of HbAS on PfEMP1-mediated phenomena. One possibility is that α^+^thalassemia selectively reduces the amount of HbS in HbAS RBCs, for example, by reducing the number of α-globins that bind β_s_ globins to form stable hemoglobin α_2_β_2_ tetramers. Opi et al. (2014) provide preliminary evidence to support this, showing that the proportion of intracellular HbS significantly decreases as the degree of α^+^thalassemia increases. How well these HbS proportions correlate with cytoadherence, rosetting, PfEMP1 expression, and “knob” densities (Cholera et al., 2008) remains to be determined. Nevertheless, this study further highlights in-vitro binding assays as valuable tools in defining how hemoglobinopathies confer malaria protection. Additional experiments using naturally-circulating isolates and hemoglobinopathic RBCs from areas where α^+^thalassemia is common are needed to confirm this study's findings, to investigate the lack of epistasis between α^+^thalassemia and HbC in clinical studies (reviewed in Taylor et al., 2012), and the effects of α^+^thalassemia and HbAS on pRBC binding to EPCR — a phenotype strongly associated with cerebral malaria syndromes that are rarely seen in HbAS children.

## Disclosure

The authors are supported by the Intramural Research Program of the NIAID, NIH, and have declared no conflicts of interest.
